# Analysis of epidermal growth factor receptor expression as a predictive factor for response to gefitinib (‘Iressa’, ZD1839) in non-small-cell lung cancer

**DOI:** 10.1038/sj.bjc.6601923

**Published:** 2004-06-08

**Authors:** H S Parra, R Cavina, F Latteri, P A Zucali, E Campagnoli, E Morenghi, G C Grimaldi, M Roncalli, A Santoro

**Affiliations:** 1 Department of Medical Oncology and Hematology, Istituto Clinico Humanitas, Via A Manzoni 56, Rozzano, Milan 20089, Italy; 2 University of Milan Medical School and Division of Pathology, Istituto Clinico Humanitas, Rozzano, Milan 20089, Italy

**Keywords:** gefitinib, ZD1839, Iressa, epidermal growth factor receptor, epidermal growth factor receptor tyrosine kinase inhibitor, lung cancer

## Abstract

Gefitinib (‘Iressa’, ZD1839) is an orally active epidermal growth factor receptor (EGFR) tyrosine kinase inhibitor that has demonstrated antitumour activity and favourable tolerability in Phase II studies. We investigated whether EGFR expression levels could predict for response to gefitinib in patients with advanced non-small-cell lung cancer (NSCLC), who received gefitinib (250 mg day^−1^) as part of a worldwide compassionate-use programme. Tissue samples were analysed by immunohistochemistry to assess membrane EGFR immunoreactivity. Of 147 patients enrolled in our institution, 50 patients were evaluable for assessment of both clinical response and EGFR expression. The objective tumour response rate was 10% and disease control was achieved in 50% of patients. Although high EGFR expression was more common in squamous-cell carcinomas than adenocarcinomas, all objective responses were observed in patients with adenocarcinoma. Response and disease control with gefitinib were not associated with high EGFR expression. Overall, median survival was 4 months, and the 1-year survival rate was 18%. Strong EGFR staining correlated with shorter survival time for all patients. Gefitinib demonstrated promising clinical activity in this group of patients with NSCLC. These results have also shown that EGFR expression is not a significant predictive factor for response to gefitinib.

The epidermal growth factor receptor (EGFR) is an important target for anticancer therapy. It is expressed or highly expressed in a variety of solid tumours, such as breast, head and neck, prostate, and non-small-cell lung cancer (NSCLC) (Salomon *et al*, 1995). Some studies have indicated that high baseline EGFR expression is associated with poor prognosis in patients with NSCLC (Volm *et al*, 1998; Ohsaki *et al*, 2000). Activation of the EGFR initiates a network of downstream pathways that are implicated in tumorigenic processes such as cell survival, proliferation, metastasis and decreased apoptosis (Wells, 2000). The design of several novel biological agents has centred on specifically inhibiting this key factor in tumour biology and encouraging clinical results have been observed.

In parallel with the promising development of anti-EGFR approaches, there has been considerable interest in examining the EGFR as a predictive factor for response to these agents. Identification of predictive factors for clinical outcome is important for all treatment strategies for NSCLC, to aid the management of this disease. Disease characteristics such as performance status (PS), histological subtype and weight loss have been investigated as possible prognostic parameters (Paesmans *et al*, 1995; Takigawa *et al*, 1996). Recent attention has also focused on a number of biological markers such as the EGFR, the tumour suppressor p53, the proliferation marker Ki67 and the apoptosis regulator Bcl-2 (Nicholson *et al*, 2001; Brundage *et al*, 2002; Martin *et al*, 2003). It is not yet clear whether the EGFR is a useful predictive factor for response to EGFR-targeted agents, and the current evidence does not support EGFR screening to select patients who would benefit from EGFR-targeted therapy (Arteaga, 2002).

The orally active EGFR tyrosine kinase inhibitor (EGFR-TKI) gefitinib (‘Iressa’, ZD1839) is a leading agent in the field of EGFR-targeted therapy. In two large Phase II trials involving pretreated patients with advanced NSCLC, gefitinib monotherapy was well tolerated and demonstrated clinically meaningful antitumour activity (Fukuoka *et al*, 2003; Kris *et al*, 2003). Objective response rates of 12–18% were observed with gefitinib 250 mg day^−1^ and over 40% of patients had disease control. In addition, symptom relief was experienced by approximately 40% of symptomatic patients. Two Phase III trials of docetaxel as second-line treatment of NSCLC reported objective response rates of 5.5–6.7%, while in another Phase III trial that compared second-line docetaxel and pemetrexed, objective response rates of 9% were observed in both arms (Fossella *et al*, 2000; Shepherd *et al*, 2000; Hanna *et al*, 2003) An Expanded Access Programme (EAP) has contributed to the extensive clinical experience with gefitinib; to date, more than 92 000 patients have been treated with this novel agent worldwide (Forsythe and Faulkner, 2003), including more than 39 000 in the EAP.

Here, we report our results with a cohort of patients who received gefitinib as part of the EAP in Italy. This investigation was designed to help resolve whether EGFR expression could be used as a predictive factor for response to EGFR-targeted agents. We evaluated whether EGFR expression levels could predict as to which patients would exhibit a response or disease control after treatment with gefitinib, and whether there was a correlation between EGFR status and survival.

## PATIENTS AND METHODS

### Eligibility and treatment

Patients were eligible for inclusion in the EAP if no other standard treatment options were available to them and if they were ineligible for clinical trials with gefitinib. Each patient was required to have adequate haematological, renal and cardiac function, to be aged ⩾18 years, and to provide written, informed consent. Oral gefitinib was supplied at a dose of 250 mg day^−1^ for an indefinite period or until disease progression or unacceptable toxicity. To be eligible for the investigation, patients must have a complete record of clinical parameters and assessment of EGFR expression.

### Clinical assessment

Objective tumour response was assessed as complete response (CR), partial response (PR), stable disease (SD) or disease progression every 6 weeks according to the International Union Against Cancer/World Health Organisation criteria (Green and Weiss, 1992). Disease control was defined as the best tumour response of CR, PR or SD, confirmed and sustained for ⩾4 weeks. Survival was assessed from the date that gefitinib treatment commenced to the date of death, and survival curves were constructed using the Kaplan–Meier method. The duration of response was calculated (in patients with CR or PR) as the time from the first observed response until documented disease progression. The duration of disease control (in patients with CR, PR or SD) was calculated from the initiation of treatment until documented disease progression. Adverse events were graded according to National Cancer Institute Common Toxicity Criteria version 2.0.

### Detection of EGFR expression

**Figure 1:**
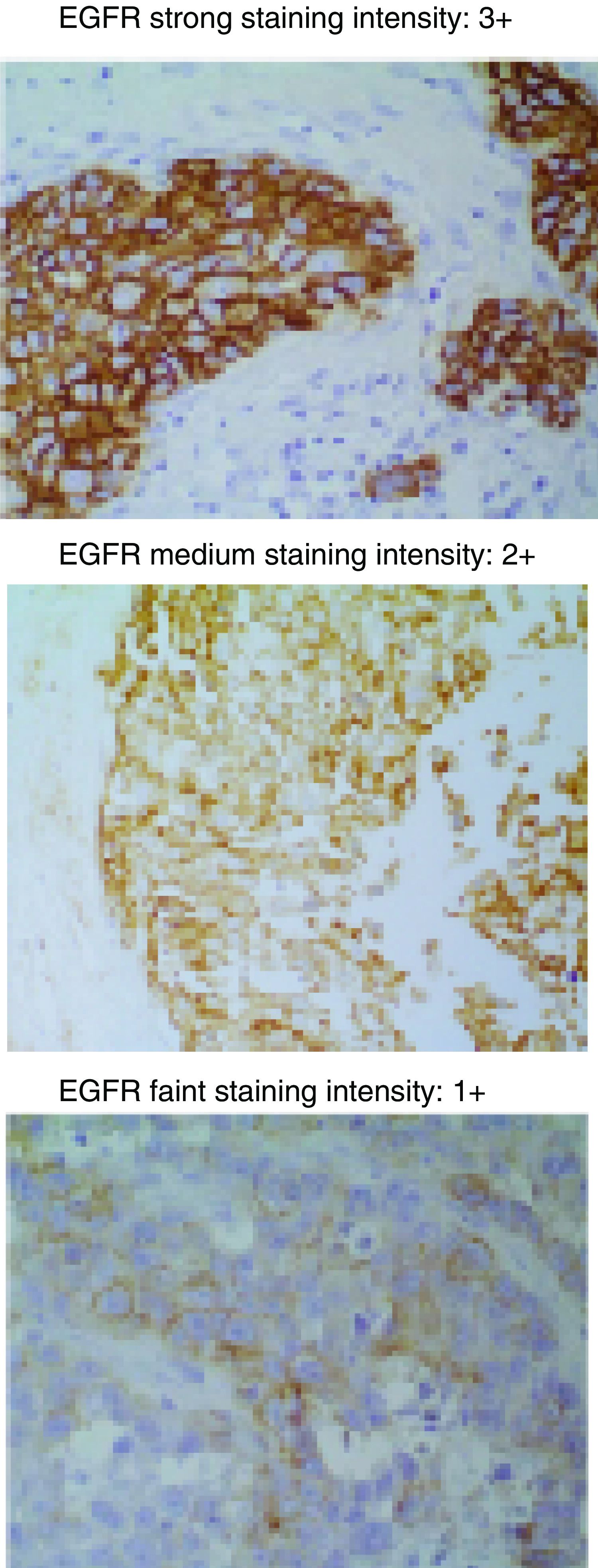
EGFR staining intensity.

Membrane EGFR immunoreactivity in paraffin-embedded tissues was analysed by immunohistochemistry using the monoclonal antibody EGFrAb-10 (clone 111.6) and the DAKO EnVision™ visualisation system. Tissue samples were classified according to the level of EGFR expression. The staining intensity of immunoreactive cells was evaluated to be negative to faint (0/1+) or medium to strong (2+/3+), as shown in Figure 1

. In addition, the percentage of neoplastic cells showing membranous immunoreactivity was evaluated semiquantitatively. Patient samples with 0–19% immunoreactive cells were classified as negative/low expressors, and those with ⩾20% immunoreactive cells were classified as high expressors. Both assessments of EGFR expression were scored blindly by two independent observers, who reached concordance for scoring in >80% of the cases. The discordant cases were scored after a consensus meeting at a double-head microscope. A *χ*^2^ test was used to determine if EGFR staining intensity correlated with baseline patient characteristics or response.

## RESULTS

### Patients

From January 2001 to May 2003, 147 patients with stage I–IV NSCLC were enrolled in the EAP at the Istituto Clinico Humanitas. In total, 50 patients were evaluable for assessment, with a complete record of clinical data and measurement of their EGFR status. This subset of patients is representative of the total patient population treated with gefitinib in our centre; baseline patient demographics are presented in Table 1

**Table 1:** Baseline patient demographics

**Characteristic**	**Evaluable patients (*n*=50)**	**Total patient population (*n*=147)**
Male : female, *n* (%)	38 : 12 (76 : 24)	110 : 37 (75 : 25)
Mean age (range), years	62 (36–80)	62 (28–82)
		
*Performance status, n* (%)		
0–1	37 (74)	117 (80)
2	13 (26)	30 (20)
		
*Stage at trial entry, n* (%)		
I–II	5 (10)	16 (11)
III–IV	45 (90)	131 (89)
		
*Histological subtype, n* (%)		
Adenocarcinoma	29 (58)	74 (50)
Squamous-cell carcinoma	9 (18)	28 (19)
Poorly differentiated carcinoma	10 (20)	27 (18)
Carcinoma NOS	2 (4)	16 (11)
Other	0	2 (1)
		
*Previous chemotherapy, n* (%)		
None	7 (14)	14 (10)
1st line	28 (56)	76 (52)
⩾2nd line	15 (30)	57 (38)
		
*Previous radiotherapy, n* (%)		
Yes	29 (58)	75 (51)
No	21 (42)	72 (49)
		
*EGFR status – staining intensity, n* (%)		
0/1+	27 (54)	n/a
2+/3+	23 (46)	
		
*EGFR status – immunoreactive cells, n* (%)		
NLE	29 (58)	n/a
HE	21 (42)	

HE=high expressor; NLE=negative/low expressor; NOS=not otherwise specified; n/a=not applicable.

. Most patients had stage III/IV disease (90%) and had previously received at least one prior chemotherapy regimen (86%).

### Clinical outcome

**Figure 2:**
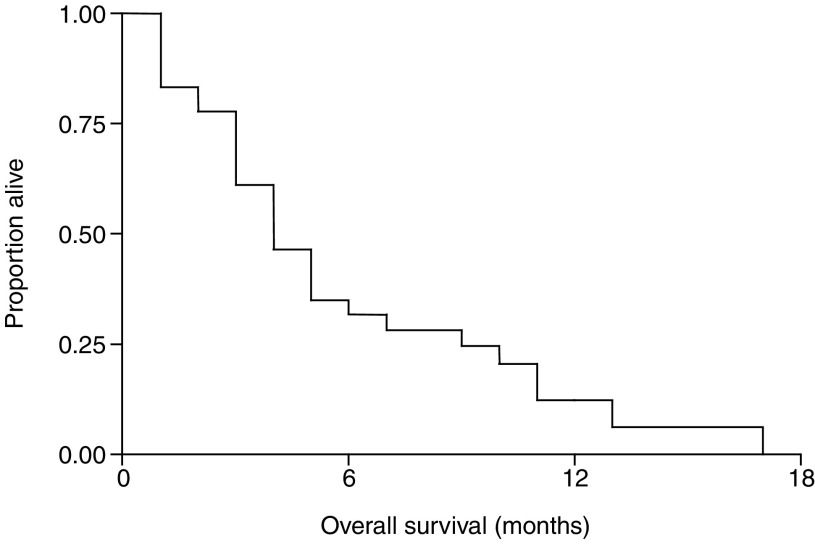
Kaplan–Meier plot showing overall survival in all evaluable patients (*n*=50).

Responses were only observed in patients with adenocarcinoma, with an overall response rate of 10% (one CR and four PR). An additional 20 patients achieved SD, amounting to a disease control rate of 50%. The median (range) durations of response and disease control were 4 (2–12) months and 6 (2–17) months, respectively. The median (range) survival was 4 (1–17) months (Figure 2

) for all evaluable patients and 9 (2–17) months in patients with disease control. The 1-year survival rate was 18%.

All patients (*n*=50) were evaluable for toxicity. Nonhaematological toxicity was mild. Grade 1/2 and grade 3 skin rash was observed in 30 (60%) and four (2%) patients, respectively. In total, 21 patients (42%) had grade 1 diarrhoea. No haematological toxicity was observed.

### EGFR expression and clinical outcome

Correlation analysis between patient characteristics and EGFR status showed that squamous-cell carcinomas were statistically significantly more likely to express EGFR than adenocarcinoma or other histological subtypes (*P*=0.009) (Table 2

**Table 2:** EGFR status according to patient characteristics

		**Staining intensity**	
	**No. of patients (*n*=50)**	**0/1+ *n* (%)**	**2+/3+ *n* (%)**
*Histology*			
Adenocarcinoma	29	20 (69)	9 (31)
Squamous-cell carcinoma	9	1 (11)	8 (89)
Other	12	6 (50)	6 (50)
		*P*=0.009^a^	
			
*Age*			
<60 years	21	7 (33)	14 (67)
⩾60 years	29	16 (55)	13 (45)
		*P*=0.12^a^	
			
*Sex*			
Female	12	4 (33)	8 (67)
Male	38	19 (50)	19 (50)
		*P*=0.31^a^	
			
*Performance status*			
0–1	37	16 (59)	21 (41)
⩾2	13	7 (54)	6 (46)
		*P*=0.57^a^	

a *χ*^2^ test.

). There was no statistically significant difference in EGFR expression related to age, sex or PS.

There was no significant correlation between response and EGFR staining intensity (*P*=0.108), or between disease control and EGFR staining intensity (*P*=0.39) (Table 3

**Table 3:** EGFR status by response and disease control

		**Staining intensity**		**Immunoreactive cells**	
	**No. of patients (*n*=50)**	**0/1+ *n* (%)**	**2+/3+ *n* (%)**	**NLE *n* (%)**	**HE *n* (%)**
*Response*					
Yes	5	1 (20)	4 (80)	2 (40)	3 (60)
No	45	26 (58)	19 (42)	27 (60)	18 (40)
		*P*=0.108^a^		*P*=0.39^a^	
					
*Disease control*					
Yes	25	15 (60)	10 (40)	17 (68)	8 (32)
No	25	12 (48)	13 (52)	12 (48)	13 (52)
		*P*=0.39^a^		*P*=0.15^a^	

a *χ*^2^ test. Response=CR+PR; Disease control=CR+PR+SD.

). Within the subset of patients with adenocarcinoma (*n*=29), there was no significant correlation between staining intensity and disease control, but there was a significant correlation between response and an EGFR status of 2+/3+ (*P*=0.009) (Table 4

**Table 4:** EGFR status and response in patients with adenocarcinoma

		**Staining intensity**		**Immunoreactive cells**	
	**No. of patients (*n*=29)**	**0/1+ *n* (%)**	**2+/3+ *n* (%)**	**NLE *n* (%)**	**HE *n* (%)**
*Response*					
Yes	5	1 (20)	4 (80)	2 (40)	3 (60)
No	24	19 (79)	5 (21)	19 (79)	5 (21)
		*P*=0.009^a^		*P*=0.07^a^	
					
*Disease control*					
Yes	17	12 (71)	5 (29)	13 (76)	4 (24)
No	12	8 (67)	4 (33)	8 (67)	4 (33)
		*P*=0.82^a^		*P*=0.56^a^	

a *χ*^2^ test. Response=CR+PR; Disease control=CR+PR+SD.

).

**Figure 3:**
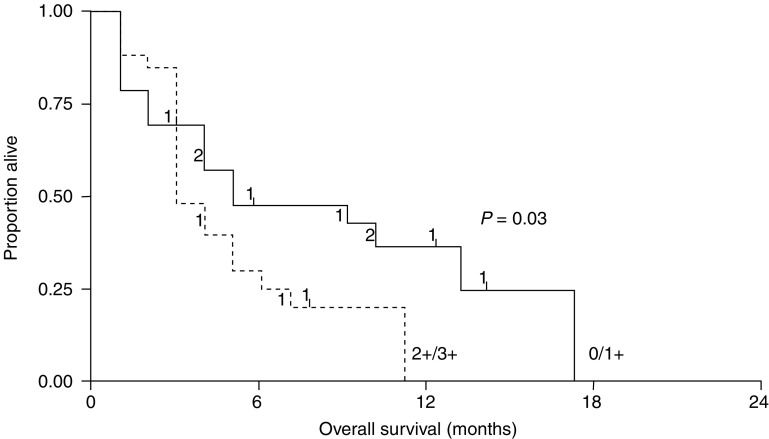
Kaplan–Meier plots showing survival according to EGFR staining intensity (*n*=50).

Evaluation of survival rates for all evaluable patients (*n*=50) showed that patients with an EGFR status of 0/1+ survived statistically significantly longer than patients with an EGFR status of 2+/3+ (*P*=0.03) (Figure 3

), which is consistent with earlier observations that high EGFR correlates with poor prognosis in NSCLC. Analysis of the patients who achieved disease control (*n*=25) revealed similar results; those with an EGFR status of 0/1+ had a longer survival than patients with an EGFR status of 2+/3+, but this difference was not statistically significant.

## DISCUSSION

For this group of 50 patients with NSCLC, who received gefitinib on a compassionate-use basis, there were promising objective tumour response and disease control rates of 10 and 50%, respectively. These results are consistent with those of two major Phase II monotherapy trials (IDEAL (‘Iressa’ Dose Evaluation in Advanced Lung cancer) 1 and 2) of gefitinib in patients with advanced NSCLC, which reported objective response rates of 11.8–18.4% and disease control rates of 42.2–54.4% (Fukuoka *et al*, 2003; Kris *et al*, 2003). Our experience in this investigation confirms that gefitinib provides valuable clinical benefit for patients with NSCLC who have no alternative treatment options.

In this series of patients treated with gefitinib, strong EGFR staining (2+/3+) correlated with shorter survival time, indicating that high EGFR expression is associated with poor prognosis. These results concur with other studies that have reported that EGFR expression assessed by immunohistochemistry is associated with shorter survival in patients with NSCLC (Volm *et al*, 1998; Ohsaki *et al*, 2000). However, a retrospective analysis of over 200 studies in different tumour types concluded that EGFR expression was a weak prognostic factor for NSCLC (Nicholson *et al*, 2001). The lack of clear consensus on this issue is partly due to the variation in EGFR detection methods used (Nicholson *et al*, 2001; Arteaga, 2002; Ciardiello and Tortora, 2003). Immunohistochemistry is arguably the most appropriate method, as this detects EGFR protein expression. However, there is currently no standardised assay in use, and differences in techniques and scoring systems prevent direct comparison between study results. The development of a standardised assay is paramount to resolving this issue.

Our results demonstrated no significant correlation between EGFR expression and either objective response or disease control resulting from gefitinib treatment. This is consistent with a recent study that assessed the correlation of EGFR membrane staining with the probability of objective response or symptom improvement resulting from gefitinib treatment in IDEAL 1 and 2. The analysis found no consistent association between EGFR expression and clinical outcome (Bailey *et al*, 2003). Objective responses or symptom relief were observed in some patients with no detectable EGFR staining, but not in other patients who had intense EGFR staining. These data suggest that tumour EGFR membrane staining is not clinically relevant for predicting response to gefitinib.

Furthermore, although patients with adenocarcinoma in this investigation were less likely to express EGFR than other histological subtypes, all objective responses were observed in patients with adenocarcinoma. Within this group there was a correlation between EGFR expression and objective response, but not disease control. High EGFR expression is more common in squamous-cell carcinomas than adenocarcinomas (Franklin *et al*, 2002), yet adenocarcinoma was identified as a potential prognostic factor in IDEAL 1 (Fukuoka *et al*, 2003). A proposed explanation has highlighted the coexpression of EGFR and high levels of HER2 in adenocarcinoma (Johnson and Arteaga, 2003). The increased potential for the formation of EGFR-HER2 heterodimers, which induce a stronger and more sustained signal than EGFR homodimers (Yarden and Sliwkowski, 2001), could result in greater reliance on this signalling network in adenocarcinoma and consequently greater sensitivity to inhibition of EGFR signalling.

As well as interactions with HER2 and other EGFR receptor family members, a number of other factors influence EGFR signalling in cancer cells, such as receptor mutations and increased expression of ligands (Arteaga, 2002; Ciardiello and Tortora, 2003). In addition, the EGFR mediates a complex network of downstream pathways that are also influenced by other signalling systems. Thus, focusing on EGFR expression levels alone might give an oversimplified view when trying to evaluate the relationship between the EGFR and response to an EGFR-targeted agent.

In conclusion, this investigation has shown that analysis of EGFR expression is not useful for the prediction of clinical outcome with gefitinib treatment in patients with advanced NSCLC. Future research should address the requirement for a standardised quantitative assay for EGFR, as well as developing assays that take further consideration of the effect of different histological subtypes on the biology of the EGFR signalling network.

‘Iressa’ is a trademark of the AstraZeneca group of companies.
